# Motivational Enhancement Interventions to Increase Pre-Exposure Prophylaxis Use in Sexual Minority Men Who Use Stimulants: Protocol for a Pilot Sequential Multiple Assignment Randomized Trial

**DOI:** 10.2196/48459

**Published:** 2023-10-13

**Authors:** Leah Davis-Ewart, Christian Grov, Rachel Verhagen, Jennifer Manuel, Michael Viamonte, Samantha Dilworth, Nicole O'Dell, Omar Valentin, Sidney Carr, Emily Cherenack, Chelsea Henderson, Susanne Doblecki-Lewis, Inbal Nahum-Shani, Adam W Carrico

**Affiliations:** 1 Health Promotion and Disease Prevention Robert Stempel College of Public Health and Social Work Florida International University Miami, FL United States; 2 Graduate School of Public Health and Health Policy City University of New York New York, NY United States; 3 Department of Psychology College of Arts and Science University of Miami Miami, FL United States; 4 School of Medicine University of California, San Francisco San Francisco, CA United States; 5 Miller School of Medicine University of Miami Miami, FL United States; 6 Institute for Social Research University of Michigan Ann Arbor, MI United States

**Keywords:** cocaine, contingency management, methamphetamine, motivational interviewing, pre-exposure prophylaxis, intervention, men, stimulant, condom, HIV testing, prevention, HIV, effectiveness, telehealth, motivation

## Abstract

**Background:**

Although pre-exposure prophylaxis (PrEP) could substantially mitigate HIV risk, sexual minority men who use stimulants commonly experience difficulties with engaging in PrEP clinical services. Motivational interviewing (MI) and contingency management (CM) reduce substance use and condomless anal sex (CAS) in this population, but these motivational enhancement interventions require modifications to promote engagement along the PrEP care continuum.

**Objective:**

PrEP Readiness Interventions for Supporting Motivation (PRISM) is a pilot sequential multiple assignment randomized trial testing the feasibility, acceptability, and preliminary effectiveness of distinct combinations of telehealth MI and CM in 70 cisgender sexual minority men who use stimulants that are not currently taking PrEP.

**Methods:**

A national sample was recruited via social networking applications to complete a baseline assessment and mail-in HIV testing. Those with nonreactive HIV results were randomized to receive either (1) a 2-session MI intervention focusing on PrEP use (session 1) and concomitant stimulant use or CAS (session 2) or (2) a CM intervention with financial incentives for documented evidence of PrEP clinical evaluation by a medical provider (US $50) and filling a PrEP prescription (US $50). At the 3-month follow-up assessment, participants who reported they had not filled a prescription for PrEP were randomized a second time to either (1) *switch* to a second-stage intervention (ie, MI+CM or CM+MI) or (2) *continue* with assessments only. Outcomes for both responders and nonresponders were reassessed at a 6-month follow-up. The primary outcome is documented evidence of filling a PrEP prescription over 6 months. Self-reported secondary outcomes include PrEP clinical evaluation by a medical provider, stimulant use, and CAS. Qualitative exit interviews were conducted with a subgroup of responders and nonresponders to characterize their experiences with the MI and CM interventions.

**Results:**

Implementation of PRISM underscores challenges in reaching sexual minority men who use stimulants to optimize HIV prevention efforts. Approximately 1 in 10 (104/1060) eligible participants have enrolled. Of the 104 who enrolled, 87 (84%) completed mail-in HIV testing. We delivered 5 preliminary HIV-positive results, including posttest counseling with referrals to confirmatory testing.

**Conclusions:**

Lessons learned from PRISM underscore the central importance of a flexible, participant-centered approach to support the engagement of sexual minority men who use stimulants. Leveraging telehealth platforms to deliver motivational enhancement interventions also expanded their reach and potential public health impact with this high-priority population. Further research is needed to determine the effectiveness of telehealth MI and CM for supporting PrEP use in sexual minority men who use stimulants.

**Trial Registration:**

ClinicalTrials.gov NCT04205487; https://clinicaltrials.gov/study/NCT04205487

**International Registered Report Identifier (IRRID):**

DERR1-10.2196/48459

## Introduction

Sexual minority men continue to account for more than two-thirds of new HIV infections in the United States [1-4], and it is estimated that 70% of seroconversions in this population occur during receptive condomless anal sex (CAS) [5-7]. Consequently, expanded efforts are needed to optimize the benefits of pre-exposure prophylaxis (PrEP) among sexual minority men at greatest risk for HIV. The Centers for Disease Control and Prevention [2,8] has estimated that 1 in 6 sexual minority men will acquire HIV in their lifetime, including half of Black sexual minority men and one-quarter of Latino sexual minority men. Over and above these profound racial disparities, there is a resurgent epidemic of methamphetamine use among sexual minority men that is disproportionately affecting Black and Latino sexual minority men [9-12]. Although methamphetamine use appeared to decline somewhat after significant public health attention in the early- and mid-2000s [13,14], it is again on the rise [13,15-19]. For over two decades, methamphetamine and other stimulant use has been identified as a prominent driver of the HIV/AIDS epidemic in sexual minority men that is associated with engagement in CAS [20,21], altered rectal immune function [22,23], and faster rates of HIV seroconversion [24-26]. The public health impact of stimulant use is evidenced by recent findings from a cohort of sexual and gender minorities who have sex with men, where 1 in 3 new HIV infections were among those reporting methamphetamine use [26].

Sexual minority men who use stimulants experience substantial barriers to navigating HIV prevention services that undermine the clinical and public health benefits of PrEP. This is evidenced by findings from one study, where sexual minority men who use methamphetamine had 5-fold greater odds of repeat prescription for postexposure prophylaxis and a 3-fold greater rate of HIV seroconversion [27]. This underscores the clear benefits of supporting the entry or re-entry of sexual minority men who use stimulants into the PrEP care continuum. Although some studies found that sexual minority men who use substances report concerns about their hypothetical ability to adhere to PrEP [28,29], actual PrEP use among sexual minority men who use substances such as methamphetamine and amyl nitrites (ie, poppers) appears to be comparable to or greater than their peers who do not use substances [30-38]. At the same time, there is growing evidence that sexual minority men who use stimulants and other substances can experience difficulties achieving prevention-effective levels of daily oral PrEP adherence [33,39,40] that may serve as early indicators of greater risk for disengagement from PrEP care and discontinuing PrEP [41-43]. For example, those engaging in heavy cocaine use have nearly 3-fold greater odds of disengagement from PrEP care compared to nonusers [41]. Taken together, there is a clear need for scalable interventions to promote the entry or re-entry of sexual minority men who use stimulants into the PrEP care continuum.

The effectiveness of motivational interviewing (MI) and contingency management (CM) interventions are supported by decades of clinical research in people with substance use disorders [44], and these are the only interventions that are efficacious for reducing both substance use and CAS in sexual minority men [45-49]. MI is an evidence-based counseling intervention targeting *intrinsic motivation* for health behavior change [50]. CM targets *extrinsic motivation* by providing tangible incentives as positive reinforcement for performing health behaviors [51], and it has also been successfully used to promote HIV-related health behavior change in people who use substances [52-57]. Interestingly, findings from one meta-analysis of the substance use disorder treatment literature indicate that MI achieves small but durable outcomes, while CM leads to moderate short-term outcomes [58]. PrEP Readiness Interventions for Supporting Motivation (PRISM) addresses an important gap by testing sequentially delivered MI and CM interventions for promoting entry or re-entry into the PrEP care continuum.

Cognitive evaluation theory proposes that motivational processes are arranged hierarchically such that the extrinsic rewards for behavior change provided during CM could paradoxically undermine intrinsic motivation in some circumstances [59]. In cognitive evaluation theory, higher-order needs for autonomy and self-determination govern the regulation of intrinsic motivation and self-efficacy, both of which could be partially undermined by CM incentives. This underscores the need for clinical research to test if there are distinct combinations of MI and CM that optimize durable PrEP use while decreasing stimulant use and CAS among sexual minority men.

**Figure 1:**
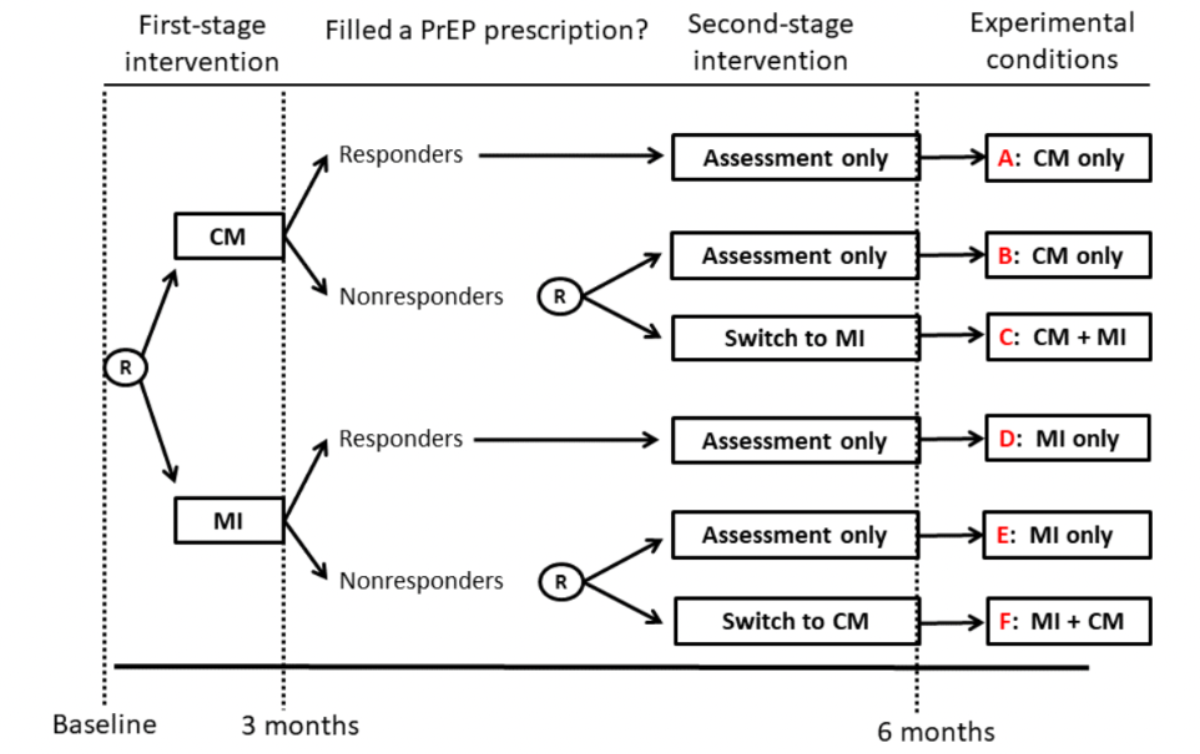
Pilot SMART of the PRISM telehealth motivational enhancement interventions to facilitate entry or re-entry into the PrEP care continuum. CM: contingency management; MI: motivational interviewing; PrEP: pre-exposure prophylaxis; PRISM: PrEP Readiness Interventions for Supporting Motivation; SMART: sequential multiple assignment randomized trial.

This paper describes the protocol for a pilot sequential multiple assignment randomized trial (SMART [60]) testing the feasibility, acceptability, and preliminary effectiveness of the PRISM telehealth motivational enhancement interventions. The primary outcome is documented evidence of filling a PrEP prescription over 6 months. As shown in Figure 1, using sequential randomization procedures allowed for a comparison of 2 first-stage interventions: MI versus CM (randomized 1:1 after baseline). Then, among those who did not fill a PrEP prescription after 3 months (ie, nonresponders), we conducted a second randomization to *switch* to a second-stage intervention (ie, MI+CM and CM+MI) versus *continue* with assessments only (also randomized 1:1 for those not on PrEP at 3 months). Because CM has been shown to yield moderate short-term effects, we hypothesize that a greater proportion of those randomized to receive CM as the first-stage intervention will have filled a PrEP prescription and report being evaluated for PrEP by a medical provider over 6 months. Because MI also targets concomitant HIV risk behaviors, we hypothesize that those randomized to receive it as the first-stage intervention will show greater reductions in self-reported stimulant use and CAS over 6 months. Finally, among nonresponders, we hypothesize that participants randomized to *switch* to a second-stage intervention (ie, CM+MI and MI+CM) will have greater improvements in the primary and secondary outcomes compared to those who *continue* with assessments only.

## Methods

### Study Design

This pilot SMART randomized 70 HIV-negative sexual minority men who use stimulants (ie, methamphetamine, powder cocaine, or crack cocaine) that are not currently taking PrEP, and completion of the final follow-up assessments is anticipated in July 2023. Participants were recruited via social networking applications across the United States. Interested individuals were directed to a web-based screener that assessed the following inclusion criteria: (1) 18 years of age or older; (2) identified as a cisgender man; (3) reported anal sex with a man (past 6 months); (4) reported using methamphetamine, powder cocaine, or crack cocaine in the past 3 months; (5) HIV-negative or unknown serostatus; (6) met Centers for Disease Control and Prevention criteria for PrEP eligibility [61]; and (7) not currently prescribed PrEP. PrEP eligibility was operationalized as any CAS regardless of partner type or any sexually transmitted infection (past 6 months).

### Research Team

Master’s and doctoral-level staff were trained in assessment administration, randomization, and intervention activities. Ongoing oversight and safety monitoring were provided by the principal investigators. Fidelity monitoring and group supervision for staff delivering MI sessions were overseen by a licensed psychologist who is a member of the Motivational Interviewing Network of Trainers. Follow-up assessments were conducted by staff who were blinded to intervention conditions. One team member with graduate-level training in qualitative methods conducted qualitative exit interviews with participants.

### Ethical Considerations

All procedures for this pilot SMART were approved by the University of Miami Institutional Review Board (UM IRB Study ID: MODCR00000105) with a reliance agreement from the City University of New York Graduate School of Public Health. All participants completed an informed consent before study enrollment. All pilot SMART activities were conducted using Zoom (Zoom Video Communications, Inc), which emerged as a flexible response to the COVID-19 pandemic and represents a scalable platform for reaching the broader population of sexual minority men who use stimulants with the PRISM telehealth motivational enhancement interventions. Potentially eligible participants were invited to an enrollment visit that included informed consent for the first 3 months of the pilot SMART and a baseline assessment via Zoom. Those who completed the enrollment visit received a US $50 Amazon gift card, Venmo, or Zelle cash application payment. In total, participants could make up to US $335 over the course of the pilot SMART, depending on the interventions they received. Information regarding the payment for each study activity is included in the paper where relevant. All data were stored on a Food and Drug Administration and Health Insurance Portability and Accountability Act–compliant University of Miami server, and all electronic files containing identifiable information were password-protected with access limited to institutional review board–approved study staff only.

### Sequential Informed Consent

At baseline, participants provided informed consent for the first 3 months of the pilot SMART with trained staff. This consent described the first-stage randomization to MI versus CM as well as the 3-month follow-up assessment. We used sequential informed consent procedures to ensure that participants did not plan to delay filling a PrEP prescription to be eligible for the second-stage randomization and the possibility of additional incentives (eg, those initially randomized to MI would delay filling a PrEP prescription to have another chance to receive CM financial incentives). After the 3-month follow-up assessment, participants completed a second informed consent that described procedures for the second-stage randomization of nonresponders, the 6-month follow-up assessment, and qualitative exit interviews. The follow-up assessments at 3 and 6 months were completed by an independent assessor who had not previously delivered MI or CM to participants. Participants were sent a link to the self-report measures to complete on their own, and they received a US $50 Amazon gift card or cash application payment for completing each assessment.

### Mail-In HIV Testing

After the baseline assessment, participants were sent a testing kit to collect a saliva sample with an OraSure HIV-1 (LOT 6691354; OraSure Technologies, Inc) testing device, with instructions on how to take the test and a prepaid return envelope to the HIV testing laboratory. Participants received a US $25 Amazon gift card or cash application payment for mailing this sample to a laboratory for HIV testing [62,63]. Participants who did not complete HIV home testing or those with reactive HIV test results were excluded prior to randomization. Participants with reactive HIV test results were provided with posttest counseling and assisted in connecting with local confirmatory testing [64].

### Pilot SMART Procedures

As shown in Figure 1, participants who provided a saliva sample that was nonreactive for HIV were randomized to a first-stage intervention: (1) a 2-session MI intervention or (2) a CM where they received up to US $100 in financial incentives for taking the first step toward starting or restarting PrEP. Randomization at baseline was stratified as a function of whether participants have previously taken PrEP with the randomization schemes created in SAS (version 9.4; SAS Institute Inc) by the data manager using randomly permuted block sizes of 2, 4, and 6. The randomization schemes were administered using REDCap (Research Electronic Data Capture; Vanderbilt University) and were not viewable by staff until the moment of randomization for each participant. Randomization probabilities at each stage were 1:1. Immediately following randomization, participants received their first MI session or a brief introduction to the financial incentives they could earn during the 3-month CM period. All participants received a US $20 Amazon gift card or cash application payment for attending this first-stage randomization visit. After 3 months, participants who reported that they have not filled a PrEP prescription in the prior 3 months are classified as nonresponders. Nonresponders were randomized a second time (no stratification) to either (1) *switch* to a second-stage intervention (MI+CM or CM+MI) or (2) *continue* with assessments only. Finally, responders and nonresponders completed a final follow-up assessment at 6 months to measure the primary and secondary outcomes. Participants received a US $50 Amazon gift card or cash application payment for completing each follow-up assessment at 3 and 6 months.

### MI Intervention

The 2-session MI intervention simultaneously targeted PrEP use (session 1) as well as co-occurring substance use and CAS (session 2). Each session was delivered approximately 1 week apart via Zoom, and participants received a US $20 Amazon gift card or cash application payment at each session. Participants had up to 3 months to complete both MI sessions.

Session 1 focused extensively on enhancing intrinsic motivation for starting or restarting PrEP. To begin, participants were invited to tell the facilitator a bit about themselves and describe what they know about PrEP. Using elicit-provide-elicit techniques, the facilitator provided relevant information to address any gaps in knowledge about PrEP. This included topics like the effectiveness and safety of PrEP, prevention effective adherence levels for sexual minority men, different oral dosing strategies (eg, 2-1-1), navigating PrEP care, and the availability of financial support for those without insurance. Then, participants were invited to describe their thoughts and feelings about the possibility of starting PrEP with selective reinforcement of change talk by the facilitator. Using the *change ruler*, participants rated on a scale of 1 to 10 how important it is for them to see a medical provider to learn more about PrEP. The facilitator used this to elicit change talk by asking participants why they did not pick a lower number (eg, “Why did you pick a 3 and not a 1?”). Next, the facilitator engaged participants in the *roadmap exercise*, where the decision regarding whether to start PrEP was presented as a fork in the road. Participants were asked to consider what life might look like in 1-2 years if they did not start PrEP and then if they chose to start PrEP. The session ended with a summary, and participants were encouraged to examine what (if any) steps they were ready to take toward starting or restarting PrEP. Those who were actively planning to start or restart PrEP were asked to elaborate on the timeline and examine any potential barriers they might encounter in accessing PrEP care.

At the beginning of session 2, participants were asked to describe their current thoughts and feelings about starting or restarting PrEP. The facilitator selectively reinforced change talk and answered any questions participants might have about navigating PrEP care. Next, participants were introduced to the focus of session 2, concomitant stimulant use, and CAS. Participants were presented with a menu of options that reflect possible topics for the session including (1) change how I use stimulants, (2) start substance use treatment, (3) attend a self-help group, (4) abstain from stimulants, (5) fewer sex partners, (6) less sex on stimulants, (7) use condoms, and (8) get tested for HIV regularly. Participants were encouraged to identify a topic of focus for the session that they wanted to discuss at greater length. Our approach to addressing stimulant use and CAS embraces the philosophy of harm reduction such that participants were not required to be ready, willing, or able to abstain from stimulant use or use condoms every time during anal sex [65]. Instead, the facilitator assisted participants with exploring a range of possible behavior change targets such as reducing the frequency of stimulant use, changing the mode of stimulant administration (eg, snorting instead of smoking), and using condoms during receptive anal sex with casual partners. Once a behavior change target was identified, participants were encouraged to elaborate on it to reinforce change talk. Using the *change ruler*, participants rated on a scale of 1 to 10 how important it is for them to make this change. The facilitator used this to elicit change talk by asking participants why they did not pick a lower number (eg, “Why did you pick a 3 and not a 1?”). Next, the facilitator engaged the participant in the *roadmap exercise*, where the decision regarding whether to make this change was presented as a fork in the road. Participants were asked to consider what life might look like in 1-2 years if they did not make a change and then if they chose to change. The session ended with a summary, and participants were encouraged to examine what steps (if any) they were ready to take toward change. Those who were actively planning to make a change in their behavior were asked to elaborate on the timeline and examine any potential barriers they might encounter.

### CM Intervention

Facilitators provided a brief overview of the CM protocol and encouraged participants to ask any questions they may have about how to access PrEP in their community. Facilitators provided tailored referrals for local PrEP services [66] and web-based PrEP providers. Participants were provided financial incentives as positive reinforcement for 2 key behaviors that are crucial to PrEP care continuum entry or re-entry. First, participants received a US $50 incentive for documented evidence of completing a medical visit for PrEP clinical evaluation that includes HIV testing. Documentation can include either a note from a medical provider indicating that the participant was seen for a PrEP evaluation or laboratory results consistent with PrEP clinical evaluation (eg, HIV testing and kidney function). Second, participants who provided evidence that they filled a recent PrEP prescription for Truvada or Descovy matched to their photo identification (eg, photo of the medication bottle), received an additional US $50 incentive. Participants on Apretude as injectable PrEP were asked to provide documentation from their provider or medical record demonstrating that they received an injection to obtain the US $50 incentive. Because filling a PrEP prescription requires participants to receive a PrEP clinical evaluation by a medical provider, those who had not previously received US $50 for a PrEP clinical evaluation received US $100 when they provided evidence they filled a PrEP prescription. To provide timely positive reinforcement for these behaviors, incentives were provided via remote payment applications (eg, Venmo and Zelle) within 2 business days.

### Fidelity Monitoring

The procedures for monitoring the fidelity of the CM and MI intervention protocols were as follows: (1) detailed curriculum manuals for each intervention, (2) intensive training in delivering MI and CM, (3) incorporation of mock training sessions to ensure staff meet performance criteria for delivery, (4) facilitator checklists, and (5) audio taping of sessions. Audio recordings of facilitator sessions were reviewed to assess adherence to the protocol, delivery, interpersonal skills, facilitator or participant rapport, and session flow. Fidelity monitoring was also crucial to mitigate the risk of contamination (eg, possible delivery of MI counseling during CM). The first 3 participants for each staff member (MI and CM) were reviewed immediately. Staff members also participated in ongoing individual and group supervisions. Fidelity ratings of MI sessions used the MI treatment integrity protocol [67], which is the gold standard approach for monitoring the fidelity of MI. MI treatment integrity scores review 4 global categories (cultivating change talk, softening sustain talk, partnership, and empathy) and are based on a Likert-scale from 1 (low) to 5 (high) [68].

### Primary Outcome

The primary outcome is verified evidence that a participant filled a PrEP prescription (ie, photo of a recently filled medication bottle) over the 6-month follow-up. For participants who did not provide evidence of a PrEP prescription as part of CM, those who reported taking PrEP were subsequently notified that they could receive an additional US $20 incentive for providing evidence of a recent PrEP prescription. We chose to implement this strategy to mitigate differential assessment of the primary outcome due to CM incentives. Participants were offered the US $20 incentive for providing evidence only after they self-reported recently filling a PrEP prescription so that it did not function as a CM incentive.

### Secondary Outcomes

Participants completed self-report measures over the 6-month follow-up to assess several secondary outcomes that are described briefly below.

#### PrEP Clinical Evaluation

Participants were asked to indicate if they attended an appointment with a medical provider about starting PrEP in the past 3 months. Those who indicated they attended any medical appointment about starting PrEP over the 6-month follow-up were classified as completing PrEP clinical evaluation.

#### Stimulant Use Severity

The World Health Organization Alcohol, Smoking, and Substance Involvement Screening Test is a validated self-report measure of substance use [69,70]. We plan to examine changes in the amphetamine-type stimulant and cocaine use composite scores, which measure the severity of symptoms for stimulant use disorders. Where participants reported using multiple stimulants at a given time point, the highest mean score was selected. We will also examine changes in clinically validated cut-points for moderate and severe stimulant use disorders using the Alcohol, Smoking, and Substance Involvement Screening Test (mild=0-3; moderate=4-26; and severe=27 or greater). Again, where participants are using multiple stimulants at a given time point, the cocaine or amphetamine-type stimulant use score that reflects higher severity was selected.

#### Receptive and Insertive CAS

Participants were asked to separately indicate the number of men with whom they had receptive or insertive anal sex “without a condom at least part of the time” in the past 3 months. If participants reported having CAS partners, they were asked to separately indicate the number of receptive and insertive CAS partners who were taking PrEP or were HIV-positive undetectable to examine the extent to which participants are biomed sorting [71].

#### PrEP Intentions

A 3-item PrEP intentions scale assessed intentions to complete certain PrEP-related behaviors within the next 3 months (eg, “During the next 3 months, I will talk to a health care provider about PrEP.”). Responses were rated on a 4-point scale from definitely will not do (1) to definitely will (4) with higher scores indicating stronger intentions to use PrEP [72].

#### PrEP Self-Efficacy

An 8-item scale assessed how difficult participants viewed behavior skills related to PrEP use (eg, “How difficult would it be for you to talk openly and honestly with a doctor about your sexual behaviors?”). Participants rated their responses on a 4-point scale ranging from very hard to do (1) to very easy to do (4) with higher ratings indicating greater self-efficacy for PrEP use [72].

#### PrEP Attitudes

A 5-item scale assessed attitudes toward PrEP use (eg, “PrEP is effective at preventing HIV.”) [72]. Participants rated response options on a 5-point scale ranging from strongly disagree (1) to strongly agree (5) with higher scores indicating more positive attitudes toward PrEP use.

#### PrEP Stigma

This 5-item scale assessed stigmatizing notions around PrEP use (eg, “People who take PrEP are promiscuous.”) [72]. Participants rated response options on a 5-point scale ranging from strongly disagree (1) to strongly agree (5) with higher scores indicating more stigmatizing views about PrEP [72].

### Intent-to-Treat Analyses

Using logistic regression, we will examine the intent-to-treat effects of first-stage randomization (ie, MI vs CM) on (1) any documented evidence of filling a PrEP prescription (primary outcome) over the 6-month follow-up and (2) any self-reported PrEP clinical evaluation by a medical provider over the 6-month follow-up. These logistic regression analyses will test our primary hypothesis that it is better to start with CM versus MI. Intent-to-treat analyses of secondary outcomes involving longitudinal trajectories of continuous measures will be tested using multilevel random coefficient models (ie, hierarchical linear modeling). These analyses will test our secondary hypotheses that those randomized to receive MI as a first-stage intervention will display a greater decrease in stimulant use severity and number of CAS partners over 6 months. Finally, exploratory analyses using the methods described earlier will be conducted among nonresponders only. We hypothesize that nonresponders randomized to *switch* to a second-stage intervention (ie, CM+MI and MI+CM) will have greater improvements in the primary and secondary outcomes compared to those who *continue* with assessments only.

### Power Analysis

Recent statistical modeling clearly demonstrates that it is inappropriate to use effect size estimates from a pilot trial for establishing effectiveness or informing subsequent power analyses [73]. Thus, the most appropriate focus of a pilot is to examine issues relevant to feasibility and acceptability, which do not require adequate statistical power. At the same time, we will examine the preliminary intent-to-treat effects of starting with MI versus CM in this pilot SMART. With 70 randomized participants, α=.05 (2-tailed), power=0.80, and 75% retention at 6 months, minimum detectable standardized mean differences for continuous outcomes ranged from 0.53 to 0.79 for within-subjects correlations *r* ranging from 0.10 to 0.80. For binary outcomes, using the same inputs as above plus small, medium, and large base rates of 10%, 25%, and 50%, respectively, raw proportion differences for *r*=0.10 ranged from 21% to 25% (standardized difference=0.55); and for *r*=0.80, the corresponding raw proportion differences ranged from 34% to 38% (standardized difference=0.80). Statistical power estimates following the second-stage randomization are dependent on the nonresponse rate.

### Qualitative Exit Interviews

After the 6-month follow-up assessment, participants were purposively selected for a qualitative exit interview examining their experiences with the PRISM telehealth motivational enhancement interventions received. Participants were purposively selected for in-depth qualitative interviews based on whether they were responders (n=10) or nonresponders (n=10) after 6 months. Participants invited to complete an in-depth qualitative interview received a US $50 Amazon gift card or cash application payment.

Interviews were conducted by a member of the study team that has not previously interacted with the participants at any point in the trial. Interviews were approximately 60 minutes. The interviewer completed ongoing training and supervision in qualitative methods and the pilot SMART objectives. The semistructured interview guide was tailored based on the responder status (responder at stage 1, responder at stage 2, or nonresponder at 6 months). Topics included likes and dislikes of PRISM telehealth interventions, views on the adequacy of study incentives, thoughts about PrEP, interest in injectable PrEP, and barriers and facilitators to PrEP uptake and adherence.

### Qualitative Analyses

Interviews were audio recorded on Zoom with the participants’ consent and transcribed verbatim. The transcripts are reviewed for accuracy and quality assurance. A general inductive approach [74] will be used to analyze themes relating to the acceptability and feasibility of the PRISM interventions. Codes will be derived inductively starting with a thorough reading of the transcripts and identification of relevant information expressed by participants. An initial codebook will be developed by the primary data analyst after a comprehensive reading of all transcripts. Next, a random sample of 5 interviews will be selected for a second data analyst to code for interrater agreement. The 2 researchers will review and discuss any discrepancies in the coding, emergent themes, and the need for refined definitions. The researchers will accept all codes where there is agreement and come to a consensus for areas of nonagreement based on their discussions; they will revise the codebook and recode the transcripts based on the revised codebook. Next, a second random sample of 5 interviews will be selected to determine interrater agreement. When the analysts reach an interrater agreement of 85%-90% in the second round of coding, the transcript codes will be finalized [75]. Agreement is defined as whether identical codes are applied to the selected text by both coders. Once interrater agreement is reached, all interviews will be coded by either one of the two analysts based on the final codebook following established guidelines for determining coding saturation [76].

## Results

Recruitment of participants via social networking applications reached a large pool of potentially eligible participants. Although 7800 people completed the web-based screener, the large number of screeners completed yielded 1060 (14%) potential participants. Of those who screened eligible, 104 (10%) participants completed a Zoom enrollment visit. As shown in Figure 1, 87 (84%) enrolled participants provided a saliva sample for mail-in HIV testing. Notably, we identified 5 (6%) new HIV infections, and these men were provided with posttest counseling as well as referrals to confirmatory testing. Of the 82 participants with a nonreactive HIV result, 70 (85%) were randomized to a stage 1 intervention in the pilot SMART.

There were no significant differences between enrolled and nonenrolled groups in race or ethnicity, age, and type of stimulants used. As shown in Table 1, 46 (44%) of enrolled participants were ethnic minorities with an average age of 38.5 (SD 8.9) years. Most enrolled participants reported only methamphetamine use in the last 3 months (n=65, 63%), followed by couse of methamphetamine and powder or crack cocaine (n=23, 22%), and then only powder or crack cocaine use in the last 3 months (n=16, 15%). Although enrolled participants were more likely to know about “on-demand PrEP” or “event-based dosing” as an alternative to daily oral PrEP, nearly three-fourths of enrolled and nonenrolled participants expressed interest in this dosing strategy.

**Table 1:** Comparison of eligible participants who enrolled versus those who did not enroll (N=1060).^a^

		Enrolled (n=104)	Not enrolled (n=956)	*P* value	
Age (years), mean (SD)		38.5 (89)	38.1 (10.1)	.69	
**Stimulant use (past 3 months), n (%)**					.76
	Powder or crack cocaine only	16 (15.4)	141 (14.8)		
	Methamphetamine only	65 (62.5)	630 (65.9)		
	Powder or crack cocaine and methamphetamine	23 (22.1)	185 (19.4)		
Interested in on-demand PrEP^b^, n (%)		77 (74.0)	714 (74.7)	.89	
On-demand PrEP is a good prevention choice for me, n (%)		74 (71.2)	671 (70.3)	.85	
It would be difficult for me to use on-demand PrEP, n (%)		9 (8.7)	101 (10.6)	.54	
**Race or ethnicity, n (%)**					.08
	Black or African American	16 (15.4)	76 (8.1)		
	White	58 (55.8)	548 (58.1)		
	Hispanic or Latino	21 (20.2)	212 (22.5)		
	Other ethnic minority	9 (8.7)	108 (11.4)		
**HIV status, n (%)**					.30
	HIV-negative	84 (80.8)	729 (76.3)		
	Unknown	20 (19.2)	227 (23.7)		
Diagnosed with a STI^c^ (past 6 months), n (%)		17 (16.4)	114 (12.0)	.20	
Any sex exchange for money or drugs, n (%)		29 (28.4)	303 (31.9)	.47	
Aware of on-demand PrEP, n (%)		73 (70.2)	575 (60.2)	.047	
Previously used on-demand PrEP, n (%)		4 (3.9)	25 (2.6)	.52^d^	

^a^A pooled *t* test was used to compare means across both groups for the continuous characteristic, age.

^b^PrEP: pre-exposure prophylaxis.

^c^STI: sexually transmitted infection.

^d^Chi-square test of proportions was used for all categorical characteristics except for this value, where Fisher exact test was used.

## Discussion

Implementation of the pilot SMART of the PRISM telehealth motivational enhancement interventions highlights the challenges associated with launching and sustaining a national recruitment campaign to achieve our target sample size. Additionally, the onset of the COVID-19 pandemic necessitated delivery of the PRISM motivational interventions using a telehealth platform. Participant enrollment, scheduling, and completing intervention activities via telehealth as well as mail-in HIV testing efforts afforded the research team various successes, challenges, and lessons learned.

Enrolling potentially eligible participants proved to be more difficult than anticipated such that only 1 in 10 who screened eligible enrolled in the pilot SMART. This reflects the challenges in optimizing HIV prevention with sexual minority men who use stimulants, a high-priority population that is often difficult to access because many are not actively seeking services. Despite the challenges we experienced in enrolling potentially eligible participants, there were no significant differences between enrolled and nonenrolled groups in race or ethnicity, age, and type of stimulants used. Enrolled participants appear to be generally representative of the broader population of sexual minority men who use stimulants that we screened from social networking applications. Future randomized controlled trials will likely require a substantial investment of time and resources for national recruitment campaigns to examine the effectiveness of the PRISM telehealth motivational enhancement interventions.

Throughout the pilot SMART, we developed and refined strategies to support the engagement of potentially eligible participants through SMS text messages and other communications that emphasized the nonjudgmental, flexible, and participant-centered approach of our team. We devoted substantial resources to enrolling participants with multiple contacts to facilitate scheduling and often multiple missed enrollment visits. Many eligible participants did not respond to multiple attempts to contact them via text; for those who did respond, our staff reported approximately two-thirds no-show rate for scheduled Zoom enrollment visits. PRISM staff implemented procedures regarding appointment reminders that included nonjudgmental language regarding follow-ups for missed appointments (eg, “Sorry we missed each other today. Happy to reschedule for another time.”) and encouraged participants to re-establish contact. This is consistent with findings from previous studies using social media to recruit sexual minority men most vulnerable to HIV acquisition [63,77] as well as SMS text messages to enhance engagement and retention [78].

The inclusion of HIV testing provided additional successes and challenges for our staff. Staff time was required to assemble and mail HIV testing kits, resending kits as needed, as well as time spent texting reminders to participants to return their saliva specimens. Throughout implementation of the pilot SMART, we refined standard operating procedures for ensuring timely completion of mail-in HIV testing and delivery of reactive HIV results. Although mail-in HIV testing with a US $25 incentive was feasible and acceptable in this pilot SMART, conducting rapid HIV self-testing during the enrollment visit over Zoom would have removed operational barriers to completing HIV testing as a prerequisite for randomization. There were routine delays of approximately 6 weeks in receiving HIV test results from the laboratory. Because nonreactive HIV results were required prior to the randomization visit, these delays may have contributed to the attrition of some otherwise eligible participants during this waiting period.

With the onset of the COVID-19 pandemic in 2020, our team revised the pilot SMART protocol to focus on the telehealth delivery of MI and CM to national sample. Instead of in-person, local sessions, we were able to enroll sexual minority men who use stimulants from over 30 states. Recruiting a national sample also expanded the reach of the pilot SMART to test the potential benefits of delivering telehealth motivational interventions in geographic regions with varying degrees of structural stigma toward sexual minority men and different levels of access to PrEP clinical services. As some of our participants were located in rural areas, many did not have a PrEP provider available within a reasonable traveling distance. This required referrals for web-based PrEP delivery options that were the only source of PrEP access for some participants. Randomized controlled trials are needed to determine whether and how geographic region moderates the effectiveness of telehealth motivational enhancement interventions with this high-priority population.

Although the development and implementation of this pilot SMART have been successful in many ways, we do recognize some limitations. First, the sample size is underpowered because our primary focus was to estimate the feasibility and acceptability of PRISM telehealth motivational interventions. A larger trial is needed to determine the effectiveness of distinct combinations of CM and MI for PrEP use as well as concomitant changes in substance use and CAS. Implementation of mail-in HIV testing also presented some challenges, including delays in the provision of saliva samples and laboratory wait times. Future research should determine the feasibility and acceptability of at-home rapid HIV testing with posttest counseling over Zoom.

Development and implementation of the pilot SMART of the PRISM telehealth motivational enhancement interventions for sexual minority men who use stimulants have afforded our team many successes and learning experiences. Our focus on modification of the MI and CM intervention protocols for telehealth delivery during the COVID-19 pandemic expanded their reach and potential public health impact. We also gained experience with implementing a national recruitment campaign that will guide appropriate resource allocation in subsequent randomized controlled trials. Implementing a waiting period prior to randomization did not meaningfully diminish our randomization rate and highlighted the operational efficiencies of leveraging rapid HIV testing that could be used in subsequent randomized controlled trials. We anticipate dissemination of estimates of feasibility, acceptability, and preliminary effectiveness from the pilot SMART with findings from qualitative exit interviews in the coming year. Lessons learned from this pilot SMART will guide more definitive trials of behavioral interventions to optimize success along the PrEP care continuum with this high-priority population of sexual minority men who use stimulants.

## Abbreviations

CAS: condomless anal sex
CM: contingency management
MI: motivational interviewing
PrEP: pre-exposure prophylaxis
PRISM: PrEP Readiness Interventions for Supporting Motivation
REDCap: Research Electronic Data Capture
SMART: sequential multiple assignment randomized trial

## Data Availability

The data sets generated during or analyzed during this study are available from the corresponding author on reasonable request.
